# Hibernation temperature-dependent *Pseudogymnoascus destructans* infection intensity in Palearctic bats

**DOI:** 10.1080/21505594.2018.1548685

**Published:** 2018-12-03

**Authors:** Natália Martínková, Jiri Pikula, Jan Zukal, Veronika Kovacova, Hana Bandouchova, Tomáš Bartonička, Alexander D. Botvinkin, Jiri Brichta, Heliana Dundarova, Tomasz Kokurewicz, Nancy R. Irwin, Petr Linhart, Oleg L. Orlov, Vladimir Piacek, Pavel Škrabánek, Mikhail P. Tiunov, Alexandra Zahradníková

**Affiliations:** a Institute of Vertebrate Biology, Czech Academy of Sciences, Brno, Czech Republic; b Institute of Biostatistics and Analyses, Masaryk University, Brno, Czech Republic; c Department of Ecology and Diseases of Game, Fish and Bees, University of Veterinary and Pharmaceutical Sciences Brno, Brno, Czech Republic; d Department of Botany and Zoology, Masaryk University, Brno, Czech Republic; e Epidemiology Department, Irkutsk State Medical University, Irkutsk, Russian Federation; f Department of Ecosystem Research, Environmental Risk Assessment and Conservation Biology, Institute of Biodiversity and Ecosystem Research, Sofia, Bulgaria; g Institute of Biology, Department of Vertebrate Ecology and Palaeontology, Wrocław University of Environmental and Life Sciences, Wrocław, Poland; h Department of Biology, University of York, York, UK; i International Complex Research Laboratory for Study of Climate Change, Land Use and Biodiversity, Tyumen State University, Tyumen, Russian Federation; j Department of Biochemistry, Ural State Medical University, Ekaterinburg, Russian Federation; k Department of Process Control, Faculty of Electrical Engineering and Informatics, University of Pardubice, Pardubice, Czech Republic; l Institute of Automation and Computer Science, Brno University of Technology, Brno, Czech Republic; m Institute of Biology and Soil Science, Far East Branch of the Russian Academy of Sciences, Vladivostok, Russian Federation; n Department of Muscle Cell Research, Centre of Biosciences, Institute of Molecular Physiology and Genetics, Slovak Academy of Sciences, Bratislava, Slovakia

**Keywords:** Chiroptera, fungal load, fuzzy regression, histopathology, thermal preference, white-nose syndrome

## Abstract

White-nose syndrome (WNS) is a fungal disease caused by *Pseudogymnoascus destructans* that is devastating to Nearctic bat populations but tolerated by Palearctic bats. Temperature is a factor known to be important for fungal growth and bat choice of hibernation. Here we investigated the effect of temperature on the pathogenic fungal growth in the wild across the Palearctic. We modelled body surface temperature of bats with respect to fungal infection intensity and disease severity and were able to relate this to the mean annual surface temperature at the site. Bats that hibernated at lower temperatures had less fungal growth and fewer skin lesions on their wings. Contrary to expectation derived from laboratory *P. destructans* culture experiments, natural infection intensity peaked between 5 and 6°C and decreased at warmer hibernating temperature. We made predictive maps based on bat species distributions, temperature and infection intensity and disease severity data to determine not only where *P. destructans* will be found but also where the infection will be invasive to bats across the Palearctic. Together these data highlight the mechanistic model of the interplay between environmental and biological factors, which determine progression in a wildlife disease.

## Graphical Abstract

**Figure:**
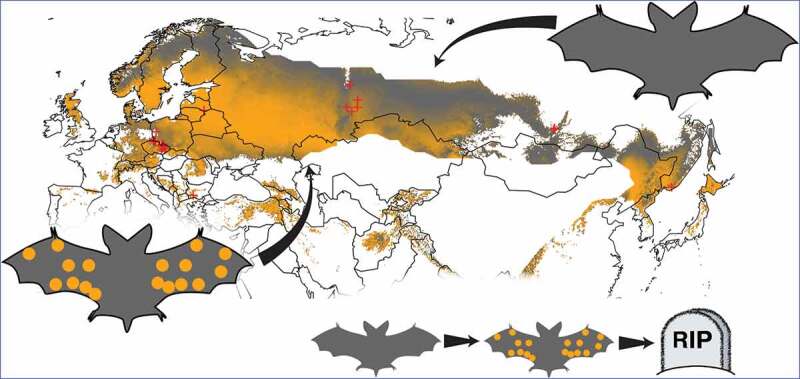

## Introduction

Hibernation as a life history trait increases overwinter survival of small mammals by reducing the risk of multiple mortality factors [1]. Emergence of white-nose syndrome (WNS) in hibernating insectivorous bats has recently compromised the benefits of hibernation and progressed into one of the most devastating wildlife risks [2–5]. Cold and moist microclimatic conditions of underground hibernacula bring together a highly virulent fungal pathogen and susceptible heterothermic hosts [6,7]. Psychrophilly of the WNS causative fungal agent, *Pseudogymnoascus destructans* [8,9], enables it to proliferate and persist long-term in contaminated hibernacula [7,10]. Invasive skin infection of bats selecting the contaminated sites for hibernation can lead to pathology progression and ultimately death of infected hosts [11–13].

Detrimental effects of WNS vary geographically and between hosts [5,14–18] as a result of alterations of disease factors that interact in enhancing or reducing mechanisms of resistance and/or tolerance to infection. While competence to control infections during torpor via immune functions is reduced [19,20], bat species may tolerate both intracellular [21] and extracellular infections [17,22]. For example, recruitment of leukocytes to the site of *P. destructans* infection was seen to be insufficient despite significant induction of gene expression of inflammatory and wound healing metabolic pathways in the infected skin of hibernating *Myotis lucifugus* bats [23,24]. In contrast to the reduced ability to overcome the infection during hibernation, aroused bats clear the *P. destructans* invasion within weeks in the early post-hibernation period [13] or develop an immunopathology response that may overwhelm the host and result in death of the diseased animal [20,21].

The duration and thermal profiles of hibernation greatly influence the activity of pathogens, the host immune response and ultimately survival [25,26]. In obligate pathogenic fungi, the specific environment of the host, including its body temperature and metabolic state, often stimulate production of secondary metabolites [25]. The secondary metabolites and proteolytic enzymes are suspected as the main virulence factors in skin infecting fungi [23,27–29]. Pathogen growth may be mitigated by the host’s choice of low hibernation temperature. Vespertilionid and rhinolophid bats of temperate regions prefer hibernation temperatures commonly ranging from 0 to 12°C [7,30]. While temperature, at which the bats hibernate, is age- and sex-specific, the mean hibernation temperature of most bat species is relatively constant, with rhinolophids selecting temperatures from the higher range [30–32]; but see [33]. Laboratory culture experiments with different *P. destructans* isolates revealed optimum temperature-dependent colony size growth between 12.5 and 15.8°C [34]. Hibernation temperatures of both Nearctic and Palearctic bat species are therefore either suboptimal for the pathogen or its growth characteristics may be different when growing on bats’ skin due to the antagonistic host-pathogen interaction. Laboratory experiments and field data suggest that temperatures of hibernation roosts influence WNS impact, in that bats survive better at lower temperatures [35–37].

Here we investigated the host-pathogen interaction at different hibernation temperatures based on quantitative measurements of infection intensity (herein fungal load and number of WNS lesions) and disease severity (WNS pathology score). To further evaluate disease severity, a new quantitative measure of fungal invasiveness scoring the ratio of the *P. destructans* biomass that has invaded living tissues compared to the total fungal load present on the wing membrane is presented. We hypothesized that in the Palearctic, where the pathogen is endemic [17], the hibernation temperature of bats influences *P. destructans* skin infection intensity. We predicted that 1) increasing bat hibernation temperature will increase the growth and virulence of the fungus and 2) bats hibernating at higher temperatures will show increased pathology in response to faster pathogen growth. Using the predictive analyses, we created spatial models estimating both infection intensity as well as disease severity across the Palearctic. Together these tools can be used in this system or others to direct resources to areas that are predicted to have the worst infection outcome.

## Materials and methods

### Study area

We sampled bats from Palearctic underground hibernacula located between 14.9° E and 133.6° E, spanning 8000 km from west to east and between 42° N and 60.1° N, encompassing 2020 km from south to north. To maximize sampling efficiency, we chose four regions interspersed from Central Europe to the Far East (Figure S1). In Europe, we report data from hibernation sites in Bulgaria (1), the Czech Republic (17), Latvia (1) and Poland (2). Ten sites sampled in Russia were located at the Ural Mts. (7), at the Lake Baikal (2) and Russian Far East (1). The sampling scheme enabled us to evaluate bats hibernating at sites with variable local climate that might influence hibernation as well as pathogen growth.

Collection of bat samples from hibernacula in the Czech Republic complied with Czech Law No. 114/1992 on Nature and Landscape Protection. Collection was based on permits 01662/MK/2012S/00775/MK/2012, 866/JS/2012 and 00356/KK/2008/AOPK issued by the Agency for Nature Conservation and Landscape Protection of the Czech Republic. The Ethical Committee of the Czech Academy of Sciences approved of all experimental procedures (No. 169/2011). The II Local Ethical Commission in Wrocław approved sampling at the “Nietoperek” Natura 2000 site in Poland (No. 45/2015). Sampling in Latvia, Bulgaria, Russia and Poland was approved by the Latvian Nature Conservation Agency (No. 3.15/146/2014-N), Bulgarian Ministry of Environment and Water (No. 645/13.08.2015 a No. 683/04.07.2016), the Institute of Plant and Animal Ecology – Ural Division of the Russian Academy of Sciences (No. 16353–2115/325) and the Regional Directorate for Environmental Protection in Gorzów Wielkopolski (No. WPN-I-6205.10.2015.AI). The authors were authorised to handle wild bats according to the Czech Certificate of Competency (No. CZ01341; §17, Act No. 246/1992 Coll.) and a permit approved by the Latvian Nature Conservation Agency (No. 05/2014).

### Data acquisition

We visited hibernacula late in the hibernation season (March-May) in 2012–2017. Prior to sampling, we measured the body surface temperature of the bat (*T*) with a Raynger MX2 non-contact IR thermometer (Raytek Corporation, USA) aimed at upper back of the bat from a distance of about 10 cm. Body surface temperature is tightly correlated with temperature of skin of a hibernating bat [38], meaning that *T* is an appropriate approximation of the temperature of bat skin. In total, we recorded *T* for 528 bats from 15 species, representing genera *Eptesicus, Murina, Myotis, Plecotus* and *Rhinolophus*. The level of fungal infection in a bat can be measured quantitatively during different stages of infection (Box 1). The fungal load that has grown colonially on the wing surface is quantified by swabbing one whole wing surface and then performing a quantitative polymerase chain reaction (qPCR) [17,39]. This is known as fungal load. The second stage of infection is when the fungus invades the skin surface forming lesions called cupping erosions that fluoresce under ultra-violet (UV) light due to the production of the secondary metabolite vitamin B_2_ [27]. These UV fluorescent lesions can be enumerated after a photograph has been taken of the wing as a relative measure of invasive infection [17,40]. Finally, there is a disease severity scoring system called histoSum, which is a semi-quantitative scoring metric of the focal pathology within the wing tissues [13]. While histoSum provides accurate WNS diagnosis and assessment of WNS pathology, it relies on destructive sampling when a wing punch biopsy is taken. To limit the harm to individual animals while retaining large sample sizes, we derived a new method that assesses the infection invasiveness by combining fungal load and number of UV fluorescent lesions.

**Box 1.** Infection intensity and disease severity measures in bats with white-nose syndrome.

**Fungal load** $\tilde{Pd}$ – Infection intensity measure approximated as the amount of *Pseudogymnoascus destructans* DNA estimated from a quantitative PCR assay [39] of a swab of the dorsal surface of an extended left wing of a bat. Units: ng cm^−2^. **Number of UV fluorescent lesions** $n_{UV}$ – Infection intensity measure estimated as a count of individual pinpoint orange-yellow dots fluorescing under UV light on the dorsal surface of an extended left wing of a bat. Units: cm^−2^. **Weighted cumulative WNS pathology score *histoSum*** – Disease severity measure estimated from histopathologic examination of a periodic-acid Schiff stained wing punch biopsy that included orange-yellow fluorescence under the UV light. The histoSum is calculated as: $histoSum=\overset{11}{\underset{i=1}{\sum }}g_{i}w_{i}$, where $g\in \left\{0,1\right\}$ means presence or absence of WNS pathology grades: surface fungal colonisation, follicle and sebaceous gland infection, single and multiple cup-like lesions, skin basement membrane breach, full thickness invasion, neutrophil infiltration, skin necrosis, skin infarction and fungal sequestration, and $w\in \left(1,2,2,6,12,13,19,20,25,30,-20\right)$ is an ordered set of weights. Values histoSum < 6 represent non-invasive *P. destructans* infection. Animals with histoSum ≥ 6 are diagnosed with WNS on histopathology. Unitless in interval $\left[-20,130\right]$. **Invasiveness** $I_{Pd}$ – Disease severity measure calculated as a ratio of tissue invasive fungal load to the total fungal load (Equation 3). Unitless in interval [0,1).

We swabbed dorsal side of the extended bat wing with a nylon (Floq Swabs, Copan Flock Technologies, Brescia, Italy) or cotton swab (Plain swab sterile plastic applicator, Copan) for laboratory examination of *P. destructans* infection and associated fungal load using qPCR. We isolated fungal DNA from swabs using QIAamp DNA Mini Kit (Qiagen, Halden, Germany) or Exgene Tissue SV plus mini Kit (GeneAll Biotechnology, Seoul, Korea) according to manufacturers’ protocol modified for swab samples. We quantified DNA from *P. destructans* with a dual-probe TaqMan (Life Technologies, Foster City, CA, USA) assay developed by Shuey, Drees [39], following the detailed protocol for the qPCR of Zukal, Bandouchova [17]. The first probe in the qPCR assay is specific to the *P. destructans* DNA, and the second probe is non-specific for *Pseudogymnoascus* fungi [39]. Samples with S-shaped curves for the genus-specific probe were classified as negative for *P. destructans* to conservatively avoid false positives [41]. To improve accuracy of fungal load estimation, we ran each sample in triplicate and each plate included triplicates of positive and negative controls for precise quantification. We estimated fungal load using cycle threshold values (*Ct*) relative to those from the positive control in each plate. We calibrated the equation depending on the *P. destructans* isolate used in the positive control from its respective dilution series calibration curve [16,41]. To remove the influence of size differences between species, we standardized the total fungal load to nanograms per 1 cm^2^ of wing area.

The wing area values originated from photographs of bat wings. We manually traced the wing membrane on at least three photographs, preferably of different individuals per species and calculated the polygon area with custom scripts in R [42] with help from packages *jpeg* [43] and *splancs* [44].

After swabbing, we photographed the bat wing over a 368 nm UV lamp three to ten times to detect lesions with yellow-orange fluorescence that are indicative of WNS [40]. In the laboratory, we manually counted the yellow-orange fluorescing WNS lesions on trans-illuminated photographs using the counting tool of ImageJ [45].

Meanwhile in the field, we used UV guidance to biopsy one WNS-suspect UV spot from each bat using a 4 mm sterile punch (Kruuse, Denmark) and we immediately fixed the tissue in 10% formalin for histopathological WNS diagnosis. We dehydrated the formalin-fixed skin samples in the laboratory, embedded them in paraffin and then prepared serial 5 μm thick sections. We visualized the fungal infection with the periodic acid-Schiff stain and examined the slides with light microscopy focusing on invasive fungal growth and identification of skin pathology grades [13]. Histological observation was under an Olympus BX51 light microscope (Olympus Corporation, Tokyo, Japan) and we used cellSense Software tools (Olympus Soft Imaging, GmbH, Münster, Germany) for measuring weighted cumulative WNS pathology score histoSum [13].

### Statistical analyses

#### A single body surface temperature measurement as a useful proxy for winter hibernation

Temperature of a bat in torpor is determined by the ambient temperature at the roost and bat thermoregulation [46,47]. The ambient temperature in underground hibernacula changes with geomorphology of the cave or mine system, its water and air flow regime and weather conditions at the site, with the greatest variation at entrances. The temperature range inside a hibernaculum is influenced by mean annual surface temperature at the site [7,48]. Although the bats change roosts throughout winter, where different roosts offer variable ambient temperature conditions at different times, data indicates that ambient temperature at the selected roosts may be stable [33]. First, we tested whether our single measurement taken during hibernation can be used as a proxy for seasonal hibernating temperatures. We used data published in Zukal, Berková [47] that report body surface temperatures measured bi-weekly between December 2002 and May 2003 in Moravian Karst, Czech Republic with a laser thermometer. To investigate seasonal stability of *T*, we excluded global outliers $T_{o}$, satisfying condition $T_{o}\subseteq T$ and $T_{o}\notin \left[Q_{1}-1.5\left(Q_{3}-Q_{1}\right),Q_{3}+1.5\left(Q_{3}-Q_{1}\right)\right]$, where *Q*_1_ and *Q*_3_ are lower and upper quartiles of *T*. We then modelled time dependence of *T* using a linear model. Body surface temperatures were considered stable during hibernation when the regression slope was statistically indistinguishable from zero.

#### Regional differences in body surface temperature of hibernating bats

Bats were considered torpid, when *T* < 13°C [7,30]. To combine the availability of ambient temperatures at the hibernaculum with roost selection of individual bats, we investigated the relationship between the mean annual surface temperature (MAST) of the hibernation site and body surface temperature of hibernating bats *T*. The MAST values were downloaded from bioclimatic dataset of the worldclim 1.4 database [49] for the geographic coordinates of the sampled sites. Given that species of bats hibernate at different temperature ranges [7,32] and species composition differs between sites [17], we chose a possibilistic fuzzy regression model rather than a statistical regression [50]. The fuzzy regression should be employed instead of statistical regression when the model is indefinite and the relationships between model parameters are vague [50,51]. The fuzzy regression can also be used when the data are hierarchically structured [52], herein implied by species composition and the respective phylogeny. Possibilistic-based fuzzy models allow describing a range of possible hibernating conditions in a climatic zone defined by MAST irrespective of the regional differences in species composition and sampling intensity. In this case, we used a fuzzy linear regression model that is given as

(1) $$\tilde{T}={\tilde{A}}_{0}+{\tilde{A}}_{1}\cdot MAST,$$

where ${\tilde{A}}_{0}$ and ${\tilde{A}}_{1}$ are fuzzy regression coefficients, and $\tilde{T}$ is a fuzzy prediction of body surface temperature. Both, the regression coefficients $\tilde{A}$ and the prediction $\tilde{T}$ are non-symmetric triangular fuzzy numbers that are described by a central value, a left and a right spread. The possibility of the central value of a fuzzy number is equal to 1, and the possibility linearly decreases down to 0 for decreasing as well as for increasing values of the fuzzy number. A range of fuzzy number values with a nonzero possibility, its support, is positively determined by the central value and by the left and the right spread [53].

To estimate the parameters $\tilde{A}$ of the fuzzy linear regression model, we used a method proposed by Lee, Tanaka [53]. The algorithm expects exact (“crisp”) observations of the dependent *T* and independent variable MAST, making the method suitable for the analysis of hibernating bat temperatures. The method combines a least squares approach (fitting of the central tendency) with the possibilistic approach (fitting of spreads) when approximating the observed linear dependence with the fuzzy linear model [53]. To improve the spread and accuracy of the model, we weighted the regression in favour of the central tendency in a ratio 5:1. We used cut-off *h* = 0.01 to signify a 1 % possibility of the value at the tails of our prediction. The used fuzzy regression method is sensitive to outliers and therefore we removed a local outlier prior to the analysis, namely the animal Mbra43RU, which was 4.5°C warmer than the next warmest animal from the same site. The fuzzy regression was fitted with an R package *fuzzyreg* [54].

#### Fungal growth and UV fluorescence on hibernating bats

We tested the differences in body surface temperature between sites and species with non-parametric tests, because data were not normally distributed and were not independent due to hierarchical structure with varying levels of relatedness between samples. We further evaluated the site and species effect on the comparisons by predicting the body surface temperature from a linear mixed model with site, species or their interaction treated as random effects using an R package *lme4* [55].

To examine the relationship between fungal load $\tilde{Pd}$ and number of UV fluorescent lesions $n_{UV}$ with body surface temperature of hibernating bats *T*, we modelled the relationship using the nonlinear Briére2 equation [56]. The Briére2 model was previously shown as the most suitable for relating *P. destructans* growth to cultivation temperature [34].

The Briére2 model was originally derived for growth rate conditional on experimental temperature, expecting positive values of the dependent variable. However, in our case, we modelled a temperature dependence of fungal load measured from wing swabs in logarithmic scale. For this purpose, we extended the model by a scaling constant $\left(b_{5}\right)$, i.e. the modified Briére2 model is given as

(2) $$y=b_{1}T\left(T-b_{2}\right){\left(b_{3}-T\right)}^{\frac{1}{b_{4}}}+b_{5},$$

where *y* is the infection intensity measured in logarithmic scale, and $b_{1},\ldots ,b_{5}$ are unknown coefficients of the modified Briére2 model. The coefficients $b_{2}$ and *b*_3_ are the lower and the upper temperature limit, when the pathogen can replicate.

We fitted two Briére2 models (Equation 2) that differed in *y*. Specifically, we expected $y=\log_{10}\left(\tilde{Pd}/w\right)$ and $y=\log_{10}\left(n_{UV}/w\right)$, where *w* is species-specific wing area in cm^2^, and $\tilde{Pd}$ is fungal load (ng) estimated with qPCR from DNA isolated from a wing swab. The previously estimated *P. destructans* growth curves after five weeks of cultivation were parametrized with $b_{2}\in \left[-14.7,0.6\right]$ [34], which lead subsequent models to use fixed $b_{2}=0$ without empirical justification [6]. Herein, we report the models for data-driven temperature interval $\left[b_{2},13\right)$. Coefficient $b_{3}$ represents the lethal temperature for *P. destructans* and was fixed to 19.8°C [34]. We fitted the same model to the number of UV fluorescent lesions $n_{UV}$. The model was fitted with the *nls* function in R for maximum of 200 iterations of the port algorithm and the starting values of $b_{1},\ldots ,b_{5}$ were estimated from iterative searches with custom scripts.

We were interested in the general response of the infection intensity measures $\tilde{Pd}$ and $n_{UV}$ to body surface temperature in each species. We estimated the unit rate of change of fungal load and number of UV fluorescent lesions with body surface temperature in each species as the slope of respective species-wise linear models. To test the sensitivity of the non-linear model to host species community composition and its accuracy in predicting the infection with sampling differences, we used a blocked cross-validation procedure [57]. In each round, we removed a block of data representing a single species, forming a testing set, and updated the model on the training set containing the data without the respective species. We predicted the fungal load $\tilde{Pd}$ and number of UV fluorescent lesions $n_{UV}$ on the testing set and we compared species-wise model performance based on the mean squared error (original model) and mean squared prediction error (models derived from the testing sets).

#### Invasiveness of P. destructans infection

Following skin surface colonization, *P. destructans* invades the skin and forms cupping erosions diagnostic of WNS [58]. A cupping erosion is a cup-shaped skin lesion densely packed with fungal hyphae [12] containing fungal cells with about 1500 nuclei [59]. The fungus in the cupping erosions produces secondary metabolites that further damage skin of the host [27]. The fungal colonization on the surface of a bat wing without pathological changes in host’s skin does not constitute development of the disease [12]. Current quantitative measures of infection intensity (fungal load, number of UV fluorescent lesions) and disease severity (histoSum) do not provide information about the fungal load that is growing invasively within the living skin tissue. We therefore derived a measure of fungal load within the tissue that will determine severity of the disease. We modelled the invasiveness of the *P. destructans* infection $I_{Pd}$ from the ratio of tissue invasive fungal growth to the total fungal growth present on the wing as

(3) $$I_{Pd}=\frac{\overset{⌣}{Pd}}{\tilde{Pd}+\overset{⌣}{Pd}}=\frac{n_{UV}\cdot {\overset{⌣}{Pd}}^{1}}{\tilde{Pd}+n_{UV}\cdot {\overset{⌣}{Pd}}^{1}},$$

where $\overset{˘}{Pd}$ is the invasive fungal load in host tissues, $\tilde{Pd}$ is fungal load on the wing surface measured with qPCR from a wing swab, $n_{UV}$ is the number of skin lesions on a bat wing with characteristic yellow-orange fluorescence under UV light and $\overset{⌣}{Pd}{\;}^{1}$ is fungal load in a single cupping erosion and is equal to 49.03 pg [59]. Invasiveness is meaningful only for animals that are positive for both $\tilde{Pd}$ and $n_{UV}$.

We tested the relationship between invasiveness *I_Pd_* and other measures of infection intensity and disease severity with segmented linear regression, utilising an R package *segmented* [60].

#### Geographical modelling

With known geographic variation in infection intensity and disease severity, we were interested in predicting the distribution of *P. destructans* infection in the Palearctic. We modelled fungal load on the wing surface, number of UV fluorescent lesions and invasiveness of fungal growth based on local MAST across the Palearctic. To keep the predictions within the limits of available data, we reduced the area of the Palearctic in two ways. First, we considered only distribution ranges of bat hosts that were sampled (Figure S1). We masked the raster spanning the extent of the Palearctic with resolution of 10'' with a combined map of distribution ranges of sampled species. Sampled species distribution ranges were downloaded from the IUCN Red List Terrestrial Mammals database [61] and the polygons with confirmed presence of the respective species were used. Prior to combining the distribution ranges, we simplified the individual range polygons with the Douglas-Peucker algorithm [62] with tolerance equal to 0.001 to fix the self-intersecting polygons in coastal areas. MAST values were downloaded from bioclimatic dataset of the worldclim 1.4 database [49], downloaded on 29 May, 2017, for the remaining raster points. We further masked raster points for which MAST values were outside of the interval from which we measured the data (Table S1). Using the predicted values of fungal load and number of UV fluorescent lesions given MAST, we calculated invasiveness of the *P. destructans* infection across the Palearctic (Equation 3). The geographical modelling was run in R with support from packages *rgdal* [63], *maptools* [64], *rgeos* [65] and *raster* [66].

#### Epidemiological triangle model

An epidemiological triangle models an interaction of *pathogen, host* and *environment* that results in a *disease*. With data on the *pathogen*, the *hosts* and the *environment* collected herein and in Pikula, Amelon [13], we were interested in the interplay of the factors that lead to manifestation of the WNS *disease*. The *pathogen* was represented by fungal load given as $\log_{10}\left(\tilde{Pd}+c\right)$, where $c=10^{-8}$ to facilitate inclusion of bats negative for *P. destructans* DNA on qPCR. The *host* susceptibility was estimated from the first principal component inferred from modified infection intensity measures, $\log_{10}\left(\tilde{Pd}+c\right)$ and $\log_{10}\left(n_{UV}+c\right)$. The number of UV fluorescent lesions $n_{UV}$ was modified similarly to fungal load for c=0.1 to include bats that were negative for UV fluorescence. The *environment* for the pathogen growth on the infected host was reflected in measured body surface temperature *T*.

To investigate the relative contribution of the three factors that promote disease outbreak, we proposed a new variant of an epidemiological triangle inspired by a ternary plot [67]. The diagram consists of an equilateral triangle where each altitude of the triangle corresponds to one factor (i.e. to the *pathogen*, the *hosts* and the *environment*), where the relative contribution of a factor is measured from the base of the altitude, where the relative contribution of the factor is equal to zero, to the opposite vertex, where the relative contribution of the factor is equal to one. The dependent variable (*disease*) is displayed in the diagram using a colour map. All three factors were scaled to a unitless interval $\left[0,1\right]$ and for each animal, the factors’ relative contribution was evaluated so that their row sums were equal to 1. The *disease* was estimated from the disease severity measures by reducing the dimensionality of the data to the first principal component from the weighted cumulative WNS pathology score histoSum and invasiveness (Equation 3). The ternary diagram was constructed in MATLAB.

## Results

### Regional hibernating bat temperature

Following exclusion of 20 outliers from the previously published dataset measuring seasonal changes [47], body surface temperature of hibernating bats remained statistically stable in the Moravian Karst (Czech Republic) between 13 December, 2002 and 2 May, 2003 (mean = 2.91°C, SD = 0.78, *n* = 216, linear model: $\alpha_{0}=2.99,\;\alpha_{1}=-\;0.0012,\;\;F_{1,214}=\;0.66,\;\;p=0.42$). This result shows that bats choose relatively stable roosts during winter and that a single body surface temperature measurement of a hibernating bat is a suitable approximation of the temperatures experienced throughout winter hibernation.

**Figure 1.**
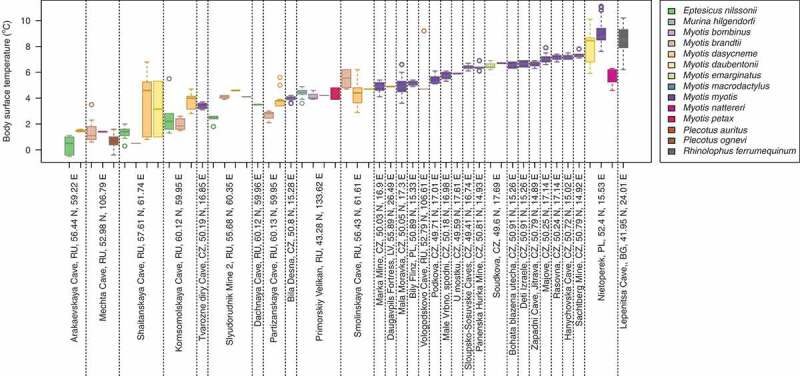
Body surface temperature of 528 bats hibernating across the Palearctic. Boxplots are ordered with increasing mean body surface temperature of all bats hibernating at the given site separated with vertical dotted lines. Bat species are colour-coded according to the legend. Body surface temperatures of two *Eptesicus nilssonii* and one *Plecotus ognevi* were in $\left[-0.5,-0.4\right]$, which does not account for freezing within the measurement error of the thermometer. BG – Bulgaria, CZ – Czech Republic, LV – Latvia, PL – Poland, RU – Russian Federation.

The body surface temperature of 528 hibernating Palearctic bats representing 15 species ranged from −0.5 to 11.1°C (mean = 5.28°C, SD = 2.35) and differed between sites (Kruskal-Wallis test: *χ*^2^ = 448.0, *df* = 30, *p* < 0.001) and species (Kruskal-Wallis test: *χ*^2^ = 301.6, *df* = 14, *p* < 0.001; Figure 1, Table S1). Out of the total of 528 animals included in the study, field and laboratory logistics resulted in 454 animals that were tested for $\tilde{Pd}$ on qPCR, 508 were photographed over UV light to estimate $n_{UV}$, and 106 were selected for histopathological examination to estimate histoSum (Table S1). Animals negative for *P. destructans* infection on qPCR hibernated at significantly lower body surface temperature (*T* = 3.2°C, *n* = 62) than animals with detectable fungal DNA (*T* = 5.6°C, *n* = 392; Kruskal-Wallis test: *χ*^2^ = 37.5, *df* = 1, *p* < 0.001). Similarly, animals with no UV fluorescent lesions hibernated at significantly lower body surface temperature (*T* = 4.1°C, *n* = 153) than animals with UV fluorescent lesions diagnostic for WNS (*T* = 5.8°C, *n* = 355; Kruskal-Wallis test: *χ*^2^ = 52.4, *df* = 1, *p* < 0.001). There was no significant difference in body surface temperature between hibernating animals positive for WNS on histopathology (*n* = 82) and those negative (*n* = 24) on histopathology (both categories: *T* = 4.8°C; Kruskal-Wallis test: *χ*^2^ = 0.04, *df* = 1, *p* = 0.84). Correcting *T* for the random effect of site and species did not influence the significance in any of the tests.

**Figure 2.**
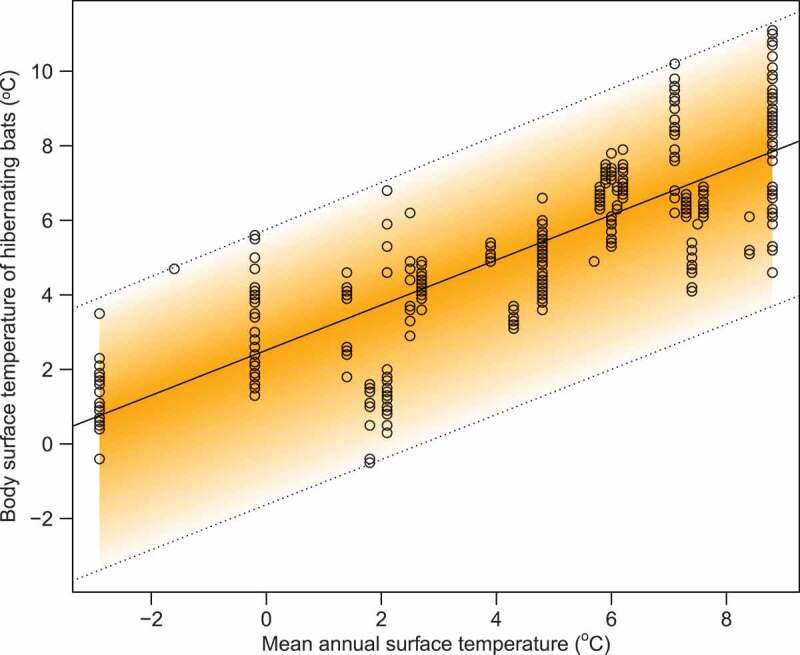
Relationship between body surface temperatures of hibernating bats dependent on mean annual surface temperature at the site. The fuzzy linear model had a fuzzy intercept with a centre equal to 2.51, left spread equal to 4.15 and right spread equal to 3.24. The fuzzy slope of the model centre is equal to 0.61, left spread equal to 0 and right spread equal to 0.03. The model shows higher body surface temperatures of hibernating bats with a slightly greater variation at warmer climates.

The relationship between the body surface temperature of hibernating bats *T* and mean annual surface temperature at the site MAST can be described by a fuzzy linear model $\tilde{T}=\left(2.51,4.15,3.24\right)+\left(0.61,0.00,0.03\right)\cdot MAST$ (Figure 2). According to the model, the central tendency of the body surface temperature is given as $T_{c}=2.51+0.61\cdot MAST$. The lower boundary of the body surface temperature is given as $T_{L}=\left(2.51-4.15\right)+0.61\cdot$*MAST*, and the upper boundary is given as $T_{U}=\left(2.51+\;3.24\right)+\left(0.61+0.03\right)\cdot$*MAST*. Thus, at the lowest recorded MAST, all hibernating bats’ body surface temperature fits in the range of $\left[-3.4,\;3.9\right]$. At the highest recorded MAST, the range of the available body surface temperatures will be greater $\left[3.7,\;11.3\right]$.

### Growth and invasiveness of P. destructans in the Palearctic

**Figure 3.**
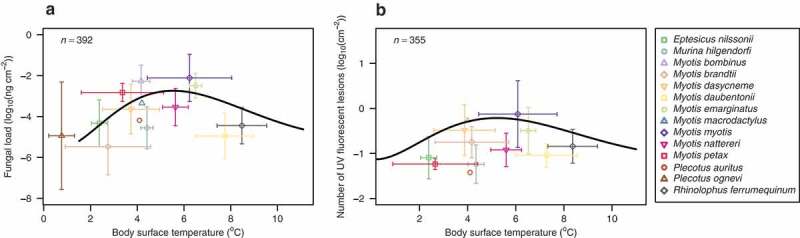
Temperature-dependent fungal load and number of invasive UV fluorescent skin lesions in hibernating bats. Plotting area limits display the measured data ranges (Table S1) with symbols representing species means and whiskers the respective standard deviations. Black lines signify the fitted model representing a non-linear regression with the Briére2 function. (a) Fungal load estimated from quantitative PCR detecting *P. destructans* DNA in wing swabs. (b) Number of UV fluorescent skin lesions on a wing of hibernating bats.

Following the estimation of the Briére2 function parameter values, minimum temperatures that predicted the limit of the infection intensity in the model (parameter *b*_2_ in Equation 2) indicated that the pathogen effectively stops replicating on a hibernating bat with T<1.5°C and further UV fluorescent lesions do not develop on bats with T<0.4°C (Table 1, Figure 3). The predicted fungal load reached a maximum on bats with a body surface temperature equal to 5.5°C. The maximum number of UV fluorescent skin lesions was predicted on bats with a body surface temperature equal to 5.1°C. The weighted cumulative WNS pathology score, histoSum, exhibited no relationship with body surface temperature of hibernating bats (Briére2 model could not be fitted).

**Table 1.** Fitted parameters of the Briére2 model (Equation 2) characterizing fungal load and UV fluorescence depending on temperature. 95 % confidence intervals for parameter estimates are given in square brackets. $\tilde{Pd}$ – surface fungal colonization, measured as fungal DNA load estimated by qPCR from a wing swab, *n_UV_* – number of UV fluorescent skin lesions diagnostic for WNS counted from wing membrane photographs trans-illuminated over a Wood’s lamp, *w* – species-specific wing membrane area, *T* – body surface temperature of a hibernating bat, MAST – mean annual surface temperature at the sampling site.

Variable		Briére2 model parameters				
dependent	independent	*b*_1_	*b*_2_	*b*_3_	*b*_4_	*b*_5_
$\log_{10}\left(\tilde{Pd}/w\right)$	*T*	9.3 × 10^−9^ [−7.3 × 10^−8^, 9.1 × 10^−8^]	1.519 [−1.4,4.5]	19.8*	0.163 [0.07,0.25]	−5.19 [−7.1,-3.3]
$\log_{10}\left(\tilde{Pd}/w\right)$	MAST	1.0 × 10^−2^ [9.2 × 10^−4^, 1.9 × 10^−2^]	−3.259 [−4.2,-2.3]	9.8 [8.8,10.8]	0.767 [0.48,1.05]	−5.24 [−5.6,-4.8]
$\log_{10}\left(n_{UV}/w\right)$	*T*	7.3 × 10^−9^ [−1.3 × 10^−7^, 1.5 × 10^−7^]	0.042 [−1.2,1.2]	19.8*	0.174 [−0.05,0.40]	−1.14 [−2.6,2.7]
$\log_{10}\left(n_{UV}/w\right)$	MAST	5.4 × 10^−3^ [1.2 × 10^−3^, 9.6 × 10^−3^]	−1.862 [−3.6,-0.1]	9.1 [8.1,10.0]	0.830 [0.47,1.19]	−1.02 [−1.3,-0.8]

* Parameter fixed according to Verant, Boyles [34].

With respect to body surface temperature of individual bat species, fungal load and number of UV fluorescent lesions were statistically stable (linear model: *p* > 0.05) in all but four species, when infection intensity changed with increasing body surface temperature (Table S2). In *Myotis daubentonii*, fungal load increased with increasing body surface temperatures (slope of linear regression: $\alpha_{1}=0.35,\;df=31,\;p=0.03$). In *M. myotis*, both fungal load and number of UV fluorescent lesions decreased at higher body surface temperatures (*M. myotis*, fungal load: $\alpha_{1}=-0.39,\;df=182,\;p<0.001$ and number of UV fluorescent lesions: $\alpha_{1}=-0.12,\;df=234,\;p<0.001$). In *Myotis brandtii*, number of UV fluorescent lesions increased with increasing body surface temperature ($\alpha_{1}=0.30,\;df=7,\;p=0.04$), but in *Myotis dasycneme*, bats with higher body surface temperature had fewer UV fluorescent lesions ($\alpha_{1}=-0.22,\;df=34,\;p=0.002$; Table S2).

The blocked cross-validation procedure showed that mean squared prediction error for fungal load modelled with the Briére2 function ranges from 0 to 5.96 between species (Table S2). The respective range of mean squared error based on the presented model (Table 1) and calculated per species was [0.00,5.32] (Table S2). The increase in mean squared prediction error was the greatest in cross-validation rounds that tested model accuracy against removal of blocks including *M. myotis, Murina hilgendorfi* and *Myotis daubentonii*.

The model predicting number of UV fluorescent lesions was stable for all cross-validation rounds with the exception of the block that included *M. myotis*. For all other species, increase from mean squared error to mean squared prediction error was less than 0.3. In the case of *M. myotis*, the Briére2 function could not be fitted on the training set without *M. myotis* bats, indicating that the relationship is influenced by strong sampling bias (Table S2).

**Figure 4.**
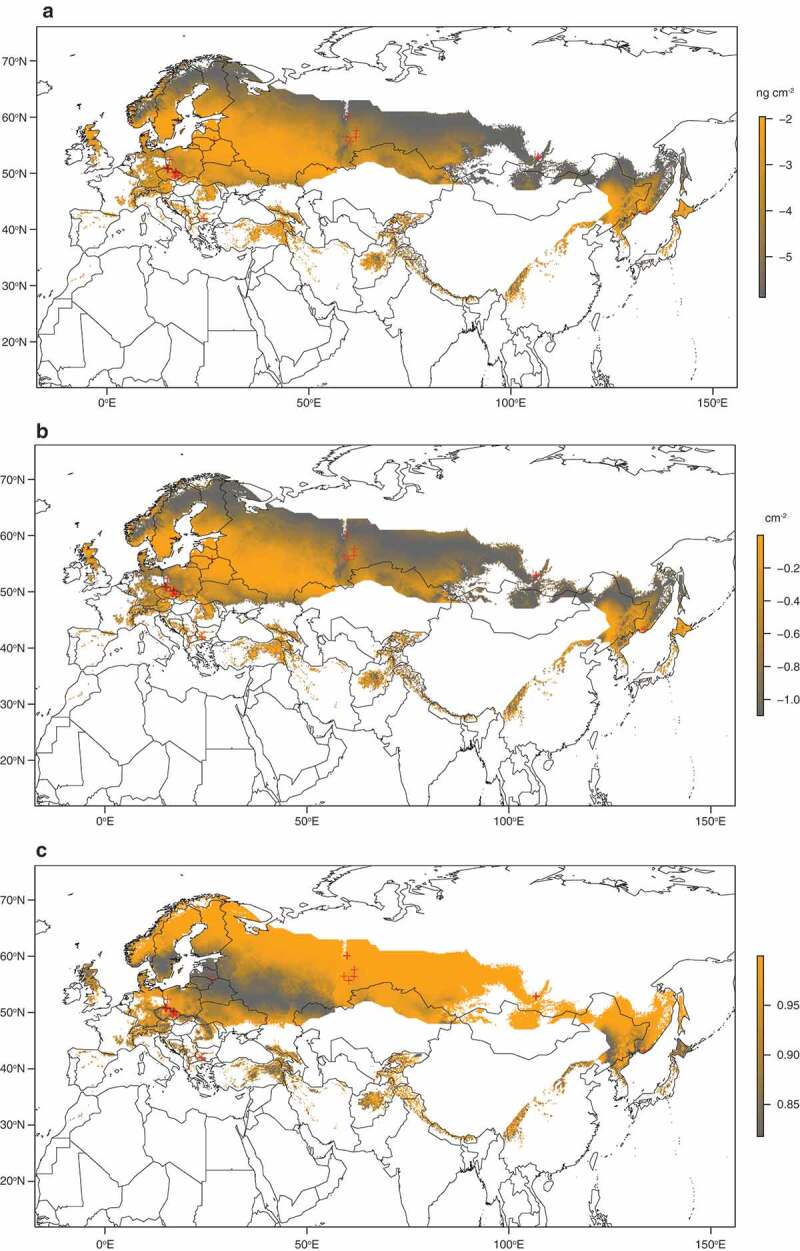
Geographic prediction of white-nose syndrome in the Palearctic. (a) Fungal load of *Pseudogymnoascus destructans* growth on hibernating bats (scale bar in log_10_(ng cm^−2^)) as a function of mean annual surface temperature at the site (°C). (b) Number of UV fluorescent skin lesions diagnostic for white-nose syndrome (scale bar in log_10_(cm^−2^)) dependent on mean annual surface temperature. (c) Invasiveness of the *P. destructans* infection (Equation 3) in hibernating bats as a ratio of invasive fungal growth based on values predicted in (b) to the sum of invasive growth and skin surface colonization based on predictions in (a). Red crosses show sampled sites and the area for prediction is delimited with distribution ranges of bat species investigated herein (Figure S1) and range of mean annual surface temperatures at the sites sampled in this study.

To predict fungal load and number of UV fluorescent skin lesions in the Palearctic, we modified the relationship in Equation 2 using MAST as the independent variable (Table 1). Both infection intensity measures showed similar geographic predictions that peaked at isocline of 5.4°C (Figure 4, Figure S1).

**Figure 5.**
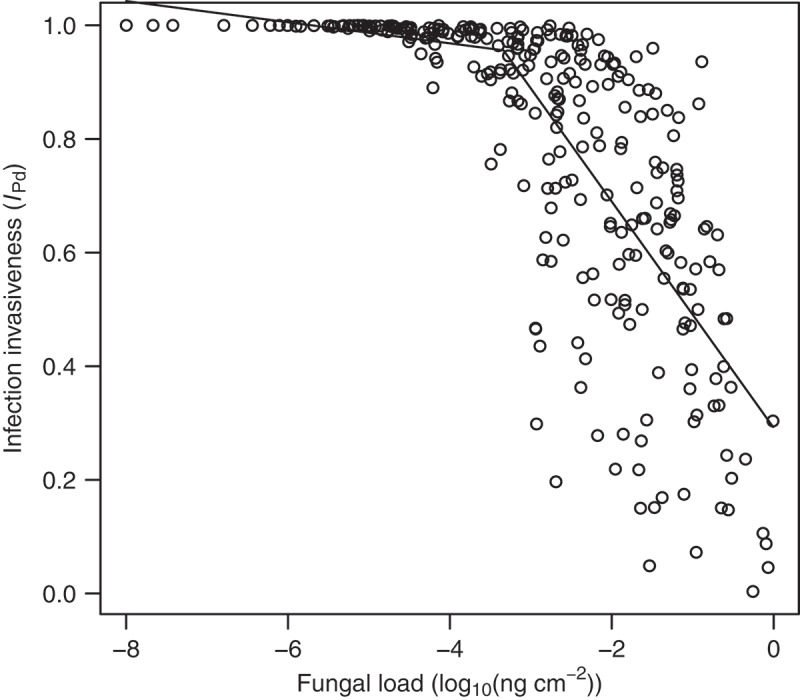
Relationship between invasiveness of *P. destructans* infection with fungal load. Invasiveness as a proportion of invasive fungal growth with respect to total fungal growth present on bat wings (Equation 3) rapidly decreases with increasing fungal load representing surface colonization after a threshold of about 0.5 pg of fungal DNA per cm^2^ of wing area of the host. The threshold was estimated from segmented linear regression (black line).

The invasiveness of the infection as a function of tissue invasive fungal growth relative to the total fungal growth differed between bat species (Kruskal-Wallis test: *χ*^2^ = 93.1, *df* = 11, *p* < 0.001) and sampling sites (Kruskal-Wallis test: *χ*^2^ = 123.5, *df* = 21, *p* < 0.001), ranging from 0.36 to 100%. The invasiveness rapidly decreased with increasing fungal load at the threshold of −3.34 (95% confidence interval: $\left[-3.75,-2.93\right]$), which corresponds to 0.5 pg of *P. destructans* DNA per cm^2^ of wing surface (segmented linear regression for fungal load less than or equal to the breakpoint $\log_{10}\left(\tilde{Pd}/w\right)\leq -3.34$: $\alpha_{0}=0.89,\;t=10.3,\;p<0.001$; $\alpha_{1}=-0.02,\;t=-1.006,\;p<0.05$ and for $\log_{10}\left(\tilde{Pd}/w\right)>-3.34$: $\alpha_{0}=0.29$; $\alpha_{1}=-0.20,\;t=-13.738,\;p<0.05$ (Figure 5). The relationship between invasiveness and number of UV fluorescent lesions *n_UV_* or the weighted cumulative WNS pathology score histoSum were indeterminate. The infection is predicted to be the least invasive (grey colour) in the mountain ranges of Europe, southern Scandinavia, British Islands and the East European Plain (Figure 4(c)).

We characterized the susceptibility of bat species to *P. destructans* infection with principal component analysis by finding a rotation of variable space given by $\tilde{Pd}$ and $n_{UV}$ that describes the greatest variance in the data. The first principal component (PC1) explained 83.7% of variance and indicated that *M. myotis* has the highest infection intensity among Palearctic bats (Figure S2(a)). The species on the opposite side of the PC1, such as *Eptesicus nilssonii* or *Barbastella barbastellus* had low fungal load and low number of UV fluorescent lesions. Ordination with disease severity measures, histoSum and invasiveness, where the PC1 explains 53% of the observed variance, showed that those are species where WNS develops into the most severe cases (Figure S2(b)).

**Figure 6.**
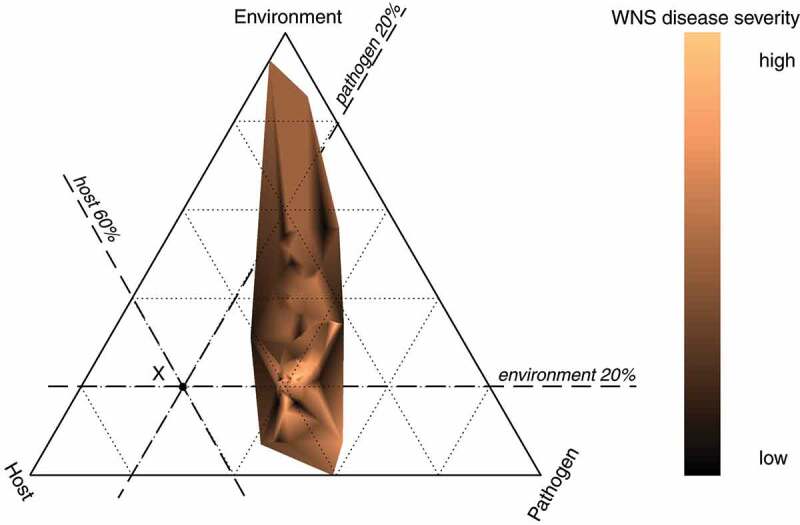
Hibernation temperature-dependent host-pathogen interaction in WNS. Epidemiological triangle based on ternary-like diagram showing relative contribution of pathogen load (Pathogen), host susceptibility (Host) and environmental suitability (Environment). A factor in the diagram is read using auxiliary lines from the edge, where the relative contribution of the factor is equal to 0, to the opposite vertex, where the relative contribution of the given factor to development of the disease reaches the value of 1. Each dotted auxiliary line indicates increments of 0.2. For example, the point X represents relative contributions of the Pathogen equal to 20 %, the Host contributed to the point X by 60 % and the Environment by 20 %. The point X is located at the white section of the triangle, meaning that the disease does not develop in our data under the conditions specified at X. The coloured polygon specifies the interaction between the Pathogen, the Host and the Environment that leads to development of the disease, with the one-dimensional representation of the WNS disease severity measures reflected in the colour gradient. Animals with the highest severity of WNS are located at the intersection of the second line from the left, meaning that the pathogen load contributed by about 40 % to disease severity, the second line from the right, meaning that the host susceptibility was similarly important to disease severity and the first line from the bottom, indicating that suitable environmental conditions contributed to disease severity by about 20 %.

We inspected the interplay between the *pathogen, host* and *environment* in development of the *disease* by constructing the epidemiological triangle for animals that were tested with all methods ($\tilde{Pd}$, $n_{UV}$, histoSum, $I_{Pd}$; *n* = 99). The epidemiological triangle displaying the relative contribution of the pathogen load, host susceptibility and environmental suitability showed that WNS develops across a wide fraction of the available parameter space with respect to the environment, i.e. a wide range of body surface temperatures of the host may lead to development of skin lesions diagnostic for WNS on histopathology (Figure 6). The relative weight of the environment ranged from 0 (horizontal edge) to 0.94 (upper vertex), indicating that the disease may develop across the available body surface temperatures of hibernating bats. The relative contribution of the pathogen to disease development is read from the left edge towards the right vertex. The pathogen contributed to disease severity by up to 0.59 with majority of values between 0.2 and 0.4. The host susceptibility, as read from the right edge towards the left vertex, contributed between 0.03 and 0.51 to disease severity. Bats with the most severe manifestation of WNS, indicated by the lightest orange colour (Figure 6), were located at the intersection where the pathogen load and the host susceptibility contributed similarly by about 40 % and the environment had low relative contribution of less than 25 %. This indicates that the interaction of the pathogen with the susceptible host is the most important interplay in developing WNS in the Palearctic, and the temperature plays a minor role once WNS skin lesions have developed in a bat.

## Discussion

### Hibernation temperatures of bats reflect individual preference in conjunction with local availability

Wild bats do not maintain universally an optimum temperature for hibernation during the whole winter [33]. However, body surface temperature data collected from *M. myotis* showed that bats in a hibernaculum chose relatively stable temperatures to roost at throughout winter [47], demonstrating that a single body surface temperature taken at the end of hibernation is a reasonable proxy for the temperature that bats had been hibernating at during winter. The stability of the body surface temperature facilitated us to model the infection intensity as expressed by the fungal load and the number of UV fluorescent skin lesions relative to body surface temperatures measured at the end of hibernation. Other species may choose different temperatures at different points in winter even within the same cave system (Figure 1), but without more data collected with temperature sensitive data-loggers on hibernating bats [38,68], we are currently at the limit of what is known. Moreover, the same species use different temperatures across their distribution range and different species choose different temperatures within one hibernaculum (Figure 1). We therefore modelled this plasticity in temperature choice by using a fuzzy regression. This enabled us to estimate the relationship between MAST, which is derived from local climate affecting the underground hibernaculum, and possible hibernating temperatures available for bats at such sites (Figure 2). Unlike statistical regression models, our fuzzy model encompasses the scope of options at microclimate level of the specific roosts, including species-specific temperature preferences.

### Temperature-dependant growth of P. destructans in the wild

Since *P. destructans* infects skin [3,11–13], body surface temperature of hibernating bats represents the temperature at which the pathogen grows. We assessed the effect of temperature on *P. destructans* infection in hibernating bats.

Our data suggest that temperature selection by hibernating bats may influence their infection status. Hibernation with low body surface temperatures significantly decreased the risk of infection, as documented by absence of both the pathogen and skin lesions from bats hibernating at about 2°C lower temperatures compared to positive bats. This is probably due to lower pathogen pressure at colder sites within the hibernaculum that, in response to the agent’s performance characteristics, become less contaminated than warmer parts. While this may be due to the bats actively avoiding conditions for *P. destructans* growth, experimental infection of bats when the bats had no thermal choice, also showed increased bat survival at lower body temperatures [35].

Taken mechanistically, we expected that in conditions when the pathogen infected the hosts, the infection intensity and disease severity induced by a pathogen that utilizes living tissues of its host would show temperature-dependent multiplication and therefore increase with increasing hibernation temperature. However, this was not what we found.

We investigated whether the same temperature constraints affect the optimum of the fungal pathogen *P. destructans* in hibernacula of different bat hosts across the Palearctic as those determined in the laboratory. The psychrophilic fungus grows optimally in the laboratory at temperatures unlikely to be encountered on a wild hibernating bat (12.5–15.8°C) [34]. We found a clear disparity between the fungal load grown on bats throughout hibernation at certain temperatures and the laboratory fungal growth curve, suggesting a shift in optimum performance of the agent under natural infection conditions. In the wild, the fungus grows on a dynamic and variable living skin tissue and under conditions that depend on the hibernaculum environment and the host microclimate roost selection. Here, contrary to expectations from easily controlled, nutrient unlimited cultivation [34], we found that the fungal load increased with increasing body surface temperature of the host and reached the highest values in bats roosting at 5.5°C (Figure 3). The bats that hibernated at higher temperatures (> 7°C) had again lower infection intensity. It may therefore be that the decrease seen in the temperature-dependant curve does not reflect a true slowing of *P. destructans* growth at these temperatures but an increased resistance to *P. destructans* in the species of bats that hibernate with higher body surface temperature. Host susceptibility differs between bat species and differences at sites contribute to regional differences in *P. destructans* infection [17,18,69]. A similar model developed for the Nearctic bats, which die of WNS more frequently than the tolerant Palearctic species [70], could help differentiate the mechanisms that influence the observations.

UV fluorescent lesions were estimated to be at the maximum at about the same predicted temperature for maximum fungal load (~ 5°C), although the relationship was strongly influenced by *M. myotis* samples (Table S2). Once established on the skin following exposure of a bat, *P. destructans* infection induced WNS pathology scores (histoSum) with no hibernation temperature-dependent pattern. Absence of pattern in disease severity with temperature would indicate that temperature is a factor to determine fungal growth when it is colonizing the surface of the wing, or invading the skin (UV fluorescent lesions), but once *P. destructans* is within the skin, the severity of the tissue damage is independent of the hibernation temperature. We assume that the physiological status of the individual bat starts to play a greater role in pathology of the invasive infection.

### Predicting geographic variation of infection intensity and disease severity

To predict geographic distribution of *P. destructans* quantitatively, we modelled infection intensity with both the estimated fungal load and number of UV fluorescent lesions based on data observed at hibernation sites together with available local climate data across the Palearctic. Both infection intensity models predicted high fungal load and number of skin lesions in European sites, decreasing towards colder climates in the north and east of Eurasia (Figure 4(a,b)). On the other hand, invasiveness of *P. destructans* infection was highest at regions with lower fungal load. There, the fungus that invaded the wing tissues contributed to the majority of the pathogen predicted to occur on a hibernating bat (Figure 4(c)). We hypothesize that the pattern will differ in the Nearctic, and invasiveness will remain high due to higher number of skin lesions in infected Nearctic bats. Low invasiveness predicted by our models in British Isles and Scandinavia corresponds well with the low levels of *P. destructans* infection data from bats from these regions [71]. Our model was restricted to MAST range from sampled sites, thus most of Western Europe was omitted from our predictions as it is warmer than the east. It would be most interesting and relevant to supplement the presented model with infection status data collected in a standardized and compatible way in countries with marginal environmental suitability for the fungus (Figure 4), and known to harbour the infection, such as France [72], Hungary [73] or Great Britain [71].

### Host resistance-susceptibility continuum

To evaluate differential susceptibility of bats to *P. destructans* infection, we proposed a new quantitative measure of fungal invasiveness distinguishing between non-invasive colonisation of the wing membrane surface and invasive damage to living skin tissues. Invasiveness is a function of both the pathogen virulence and mechanisms that protect the host exposed to the agent. The continuum of the host-pathogen interaction may range from resistance to susceptibility. Resistant bat populations will show minimum invasiveness combined with low total pathogen loads and low infection prevalence [18,74,75]. On the other hand, pathogen loads in susceptible bats will be associated with high tissue-invaded fungus and high prevalence of infection in the population [11,17]. The dichotomous infection outcome in terms of survival or mortality in the susceptible bat populations can be attributed to disease tolerance due to longer-term host-pathogen co-evolution in regions of pathogen endemicity [17,41]. Our data provide evidence that the pattern of disease invasiveness is both host species- and hibernaculum-specific, suggesting that geographic variation of all components of the epidemiological triangle plays a role in *P. destructans* infection progression (Figure 6).

### Hibernation temperature-dependant host-pathogen interaction model

**Figure 7.**
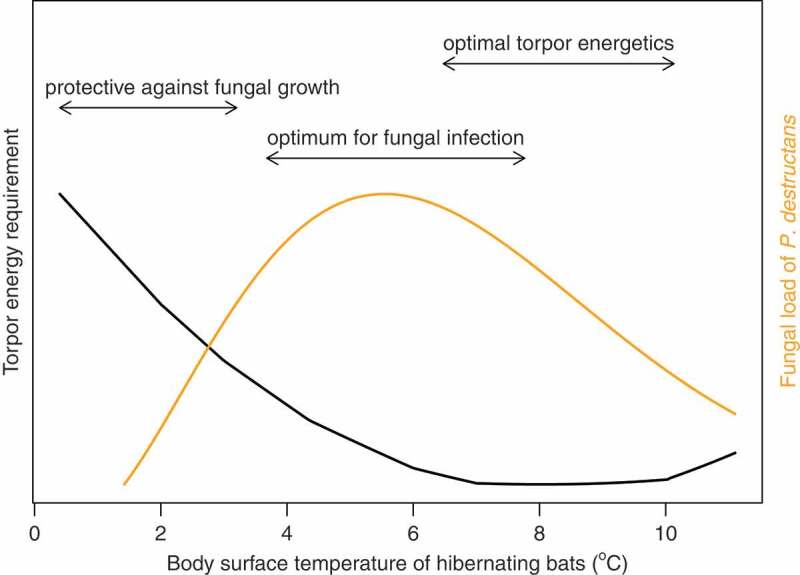
Hibernation temperature-dependant host-pathogen interaction model. The bats may influence the detrimental effects of *P. destructans* infection in two alternative choices of hibernating temperatures by either selecting low temperatures where the pathogen growth is limited or by optimizing torpor energetics. Black line – the mean of energetic models derived from literature for Palearctic bats [6], orange line – temperature-dependent Briére2 model for *P. destructans* (Table 1). The arrows correspond from left to right to minimum of data range to mean body surface temperature when bats negative for *P. destructans* on qPCR were found, 80% performance breadth for the Briére2 model, and lower 3% from the mean bat energy requirement for torpor.

We have shown that once WNS develops in the host skin, pathology progresses irrespective of the body surface temperature. In fact, host susceptibility in combination with the pathogen drives WNS severity with minor contribution of the environmental conditions (Figure 6). Presence of *P. destructans* in a hibernaculum may act as a selection pressure on bats to choose different hibernating temperatures in order to avoid excessive infection. However, the temperature that bats choose to roost at is directly related to the energy expenditure of the animals throughout the winter [46] (the model by Humphries, Thomas [46] shown as black line in Figure 7). Exposure to *P. destructans* dramatically increases winter energetics of Nearctic bats [76], compromising the evolved temperature-energy balance of infected hibernating bats. Our results indicate that the shift towards colder hibernating temperatures, where the bats are assumed to defend their body temperature [46], might be adaptive in contaminated hibernacula. While the immune competence is reduced in hibernating bats [77], their temperature choice might represent a protective mechanism to minimize pathogen pressure (Figure 7).

### Management implications

Here we examined the host-pathogen system of *P. destructans* at different ecological scales represented by individual bat species, site-specific bat communities and the landscape level across the Palearctic temperate zone of bat hibernation. We show that the presence and intensity of *P. destructans* infection is constrained by thermal choice of bats during hibernation, peaking surprisingly between 5 and 6°C, i.e. less than half the optimum temperature range expected from laboratory culture experiments with pathogenic *P. destructans* isolates. The newly proposed measure of fungal invasiveness provides evidence that no Palearctic bat species is fully resistant to *P. destructans* infection. Our predictions show that WNS is widespread across the Palearctic with varying local infection intensity and disease severity. The management of hibernacula with respect to minimizing the bat exposure to *P. destructans* should focus on conservation of sites where the bats may choose roosts with variable microclimate.

In general, our research utilizing quantitative data to analyse and model geographic distribution of *P. destructans* infection has demonstrated that the growth of the pathogenic agent on hibernating bats represents an interplay of environmental and biological factors, with temperature being a suitable environmental predictor for infection status and intensity. This approach could be useful to study other temperature-dependent host-pathogen systems such as chytridiomycosis in amphibians and spring viremia of carp in cyprinid fish.

## Supplementary Material

Supplemental Material

## Data and materials availability

All data needed to evaluate the conclusions in the paper are present in the paper and/or the Supplementary Online Materials.
