# Supplementary material for: Hibernation temperature-dependent Pseudogymnoascus destructans infection intensity in Palearctic bats
Source: Virulence. 2018 Dec 3;9(1):1734–50. doi: 10.1080/21505594.2018.1548685 (PMC10022473; doi:10.1080/21505594.2018.1548685)
Supplement: Supplemental Material [file KVIR_A_1548685_SM0063.zip › FigS2-180226biplots.pdf]

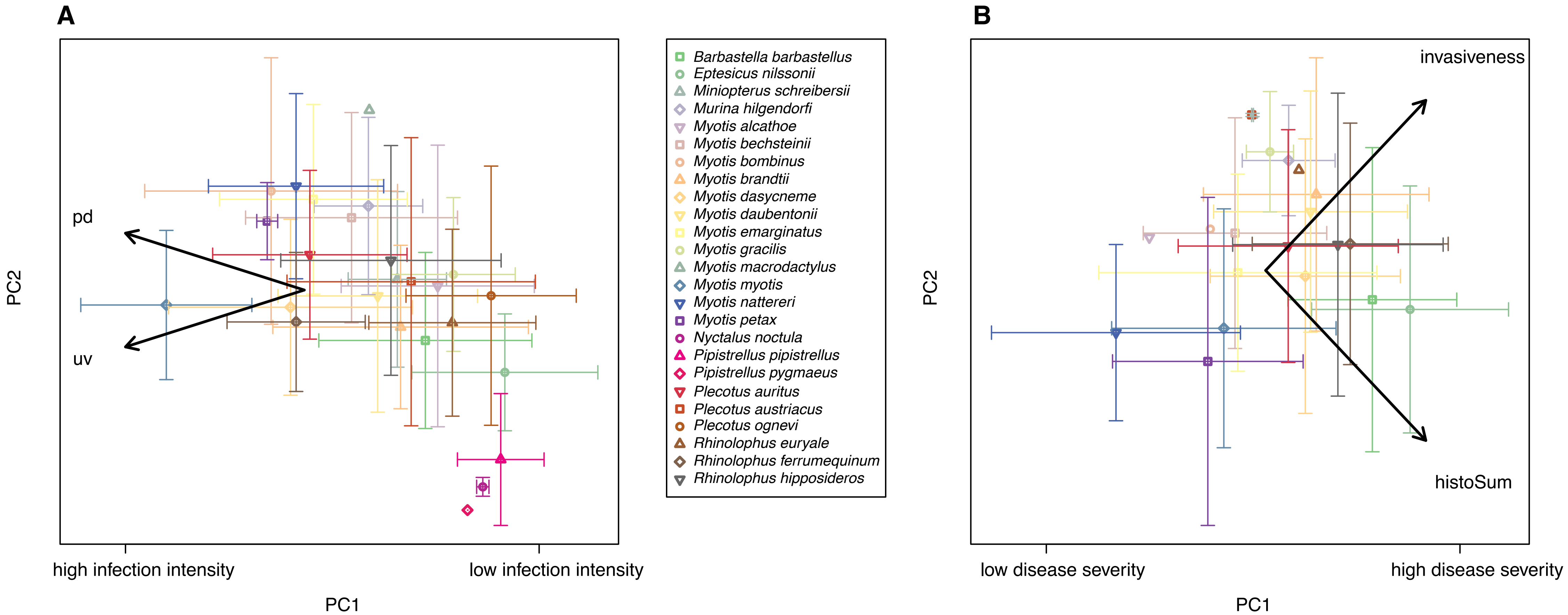
A
pd
uv
PC2
high infection intensity
low infection intensity
PC1
Barbastella barbastellus
Eptesicus nilssonii
Miniopterus schreibersii
Murina hilgendorfi
Myotis alcathoe
Myotis bechsteinii
Myotis bombinus
Myotis brandtii
Myotis dasycneme
Myotis daubentonii
Myotis emarginatus
Myotis gracilis
Myotis macrodactylus
Myotis myotis
Myotis nattereri
Myotis petax
Nyctalus noctula
Pipistrellus pipistrellus
Pipistrellus pygmaeus
Plecotus auritus
Plecotus austriacus
Plecotus ognevi
Rhinolophus euryale
Rhinolophus ferrumequinum
Rhinolophus hipposideros
B
invasiveness
histoSum
PC2
low disease severity
high disease severity
PC1
