# Supplementary material for: Hibernation temperature-dependent Pseudogymnoascus destructans infection intensity in Palearctic bats
Source: Virulence. 2018 Dec 3;9(1):1734–50. doi: 10.1080/21505594.2018.1548685 (PMC10022473; doi:10.1080/21505594.2018.1548685)
Supplement: Supplemental Material [file KVIR_A_1548685_SM0063.zip › TableS2-181101.pdf]

Hibernation temperature-dependent *Pseudogymnoascus destructans* infection intensity in Palearctic bats

Natália Martínková[1,2], Jiri Pikula,[3]* Jan Zukal,[1,4] Veronika Kovacova,[3] Hana Bandouchova,[3] Tomáš Bartonička,[4] Alexander D. Botvinkin,[5] Jiri Brichta,[3] Heliana Dundarova,[6] Tomasz Kokurewicz,[7] Nancy R. Irwin,[8] Petr Linhart,[3] Oleg L. Orlov,[9,10] Vladimir Piacek,[3] Pavel Škrabánek,[11,12] Mikhail P. Tiunov,[13] Alexandra Zahradníková, Jr.[14]

Table S2. Blocked cross-validation of the Briére2 model assessing model accuracy with respect to host species community composition. Mean squared error was calculated from residuals of the respective Briére2 model (Table 1) per species. Mean squared prediction error was evaluated from blocked cross-validation where data on all individuals of the given species were removed and the model updated on the reduced, training dataset. The reported values reflect residuals from predictions for the removed block. Rate of change shows species-specific unit change in the infection intensity measure with increasing temperature. Values without asterisks did not statistically differ from zero at $\alpha = 0.05$. NA – not available, MSE – mean squared error, MSPE – mean squared prediction error, * - $p < 0.05$, ** - $p < 0.01$, *** - $p < 0.001$.

	Fungal load				Number of UV fluorescent skin lesions			
Species	*n*	MSE	MSPE	Rate of change with *T* [range]	*n*	MSE	MSPE	Rate of change with *T* [range]
Eptesicus nilssonii	5	1.557	1.570	-2.54 [1.8,2.6]	5	0.402	0.495	0.21 [1.8,2.6]
Murina hilgendorfi	36	3.539	4.840	0.65 [3.6,4.9]	12	1.146	1.295	0.02 [3.6,4.8]
Myotis bombinus	3	0.925	0.946	1.80 [3.9,4.6]	1	0.0	0.0	NA
Myotis brandtii	12	1.606	1.672	0.19 [2.1,6.2]	9	0.284	0.303	0.30* [3.9,6.2]
Myotis dasycneme	42	1.949	2.158	-0.001 [1.2,6.2]	36	0486	0.715	-0.22** [1.4,6.2]
Myotis daubentonii	33	4.167	5.201	0.35* [4.7,10.1]	17	0.515	0.580	0.06 [4.7,8.7]
Myotis emarginatus	7	0.451	0.462	-0.51 [6.2,6.9]	5	0.256	0.258	-0.11 [6.2,6.9]
Myotis gracilis	28	5.279	5.963	-0.17 [0.6,4.7]	4	0.332	0.339	-0.003 [0.6,4.7]
Myotis macrodactylus	1	0.093	0.094	NA	0	NA	NA	NA
Myotis myotis	184	1.884	5.742	-0.39*** [3.6,11.1]	236	NA	NA	-0.12*** [3.1,11.0]
Myotis nattereri	12	1.364	1.426	-0.16 [4.6,6.3]	7	0.579	0.608	-0.20 [4.6,6.3]
Myotis petax	3	2.804	2.981	-0.18 [1.4,4.8]	2	0.455	0.480	0.07 [1.4,3.9]
Plecotus auritus	1	1.195	1.209	NA	1	1.328	1.345	NA
Plecotus ognevi	3	5.320	5.936	-4.36 [0.4,1.4]	0	NA	NA	NA
Rhinolophus ferrumequinum	22	1.514	1.757	-0.08 [6.2,10.2]	20	0.220	0.261	-0.06 [6.2,9.8]
