# Supplementary figures and images for: Hibernation temperature-dependent Pseudogymnoascus destructans infection intensity in Palearctic bats
Source: Virulence. 2018 Dec 3;9(1):1734–50. doi: 10.1080/21505594.2018.1548685 (PMC10022473; doi:10.1080/21505594.2018.1548685)

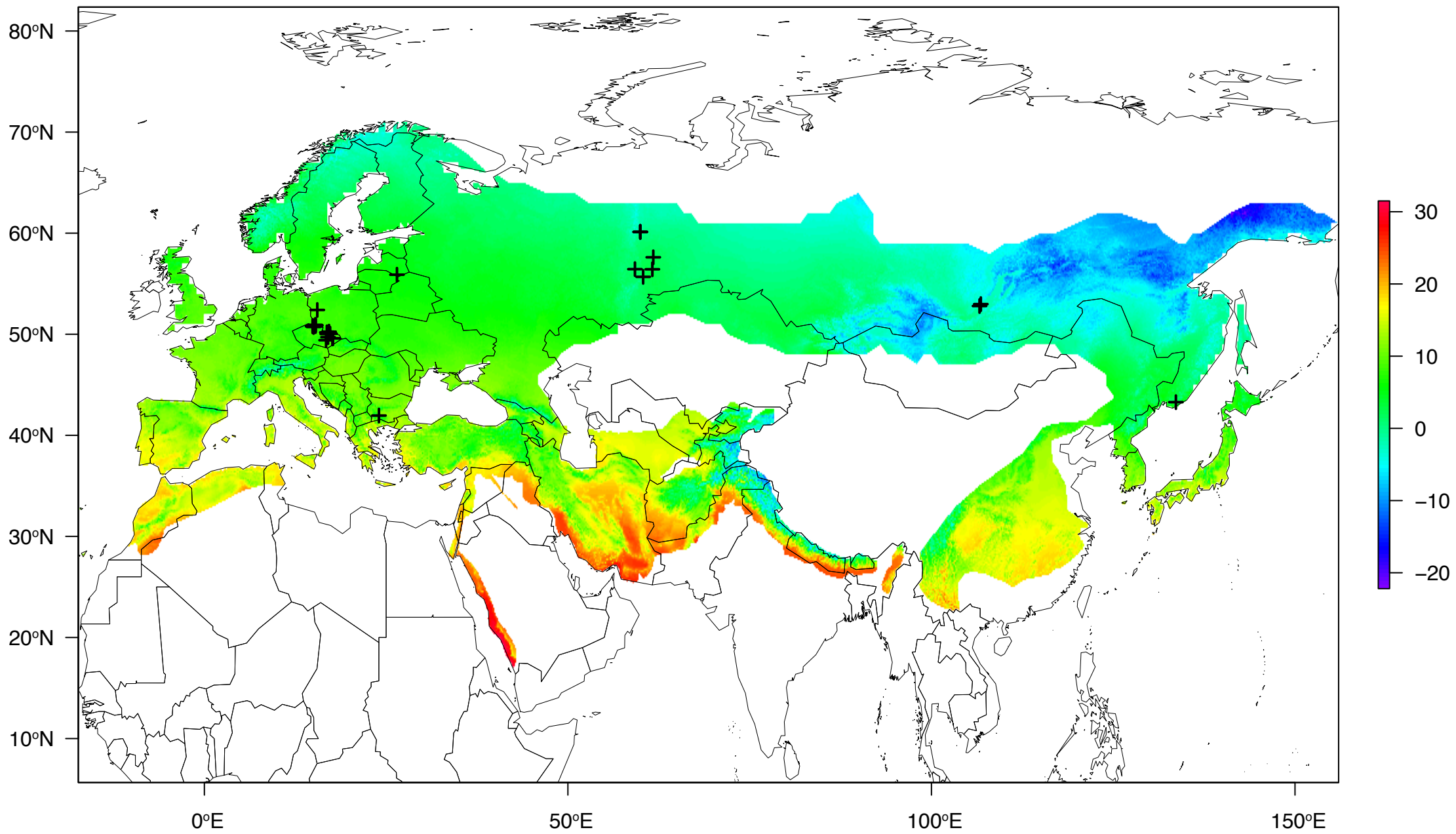

Supplement: Supplemental Material [file KVIR_A_1548685_SM0063.zip › FigS1-180227world.pdf]
